# Prognostic nomogram of overall survival for radiation therapy in hepatocellular carcinoma: a population study based on the SEER database and an external cohort

**DOI:** 10.3389/fonc.2024.1371409

**Published:** 2024-09-02

**Authors:** Lijun Chen, Qiaoyuan Wu, Jia Fu, Mengjie Jiang, Jialin Qiu, Jiaomei Tao, Litong Lin, Shenshen Chen, Yi Wu, Zhengqiang Yang, Jianxu Li, Shixiong Liang

**Affiliations:** ^1^ Department of Radiation Oncology, Guangxi Medical University Cancer Hospital, Nanning, China; ^2^ Department of Interventional Therapy, National Cancer Center/National Clinical Research Center for Cancer/Cancer Hospital, Chinese Academy of Medical Sciences and Peking Union Medical College, Beijing, China

**Keywords:** hepatocellular carcinoma (HCC), nomogram, LASSO regression, radiation therapy (RT), SEER database, overall survival (OS)

## Abstract

**Purpose:**

Radiotherapy (RT) plays an important role in the treatment of hepatocellular carcinoma (HCC). To screen patients who benefit most from RT, a nomogram for survival prediction of RT based on a large sample of patients with HCC was created and validated.

**Methods:**

A total of 2,252 cases collected from the Surveillance, Epidemiology, and End Results (SEER) database were separated into a training or an internal validation cohort in a 7:3 ratio (*n* = 1,565:650). An external validation cohort of cases from our institute was obtained (*n* = 403). LASSO regression and Cox analyses were adopted to develop a nomogram for survival prediction. The decision curve analysis (DCA), calibration curve, and time-dependent receiver operating characteristic curves (TROCs) demonstrated the reliability of the predictive model.

**Results:**

For patients with HCC who received RT, the analyses revealed that the independent survival prediction factors were T stage {T2 vs. T1, hazard ratio (HR) =1.452 [95% CI, 1.195–1.765], *p* < 0.001; T3 vs. T1, HR = 1.469 [95% CI, 1.168–1.846], *p* < 0.001; T4 vs. T1, HR = 1.291 [95% CI, 0.951–1.754], *p* = 0.101}, N stage (HR = 1.555 [95% CI, 1.338–1.805], *p* < 0.001), M stage (HR = 3.007 [95% CI, 2.645–3.418], *p* < 0.001), max tumor size (>2 and ≤5 vs. ≤2 cm, HR = 1.273 [95% CI, 0.992–1.633], *p* = 0.057; >5 and ≤10 vs. ≤2 cm, HR = 1.625 [95% CI, 1.246–2.118], *p* < 0.001; >10 vs. ≤2 cm, HR = 1.784 [95% CI, 1.335–2.385], *p* < 0.001), major vascular invasion (MVI) (HR = 1.454 [95% CI, 1.028–2.057], *p* = 0.034), alpha fetoprotein (AFP) (HR = 1.573 [95% CI, 1.315–1.882], *p* < 0.001), and chemotherapy (HR = 0.511 [95% CI, 0.454–0.576], *p* < 0.001). A nomogram constructed with these prognostic factors demonstrated outstanding predictive accuracy. The area under the curve (AUC) in the training cohort for predicting overall survival (OS) at 6, 12, 18, and 24 months was 0.824 (95% CI, 0.803–0.846), 0.824 (95% CI, 0.802–0.845), 0.816 (95% CI, 0.792–0.840), and 0.820 (95% CI, 0.794–0.846), respectively. The AUCs were similar in the other two cohorts. The DCA and calibration curve demonstrated the reliability of the predictive model.

**Conclusion:**

For patients who have been treated with RT, a nomogram constructed with T stage, N stage, M stage, tumor size, MVI, AFP, and chemotherapy has good survival prediction ability.

## Introduction

Of the total primary liver cancers, 78%–85% are hepatocellular carcinomas (HCCs). Among all malignant diseases, HCC has the third highest fatality rate worldwide (1). Treatments for patients with HCC include surgery, radiation therapy (RT), radiofrequency ablation, chemotherapy, immunotherapy, biological therapy, and arterially directed therapies. Despite the current therapeutic options, patients with HCC continue to have a high mortality and poor prognosis (2–4). Liver resection and transplantation are preferred therapies if feasible, especially for patients with suitable tumor location and reserved adequate liver. About 70% of patients with HCC are ineligible for liver resection and transplantation at first diagnosis because they have a bad performance status, late staging, or poor liver function (5). Most patients require combined treatments.

RT now is commonly used as a locoregional therapy for HCC, especially external beam radiation therapy (EBRT). It is currently the most commonly used radiotherapy technique in the clinical radiotherapy department. RT has been regarded as a standard therapeutic approach for HCC (6, 7). Better local control is achieved with stereotactic body radiation therapy (SBRT), which also results in a shorter treatment period and lower expenses (8). Hypofractionated three-dimensional conformal RT can result in an overall survival (OS) rate of 65% at 1 year (9). RT is suggested as the typical form of treatment for HCC by the National Comprehensive Cancer Network guidelines (10).

The primary dose-limiting toxicity of RT is radiation-induced hepatic toxicity (RIHT) (11). Advances in imaging and RT technologies have significantly enhanced the precision and conformity of RT. RT has become an essential and effective component of HCC treatment, particularly for patients with poor Child–Pugh scores or portal vein tumor thrombosis (12, 13). The combination of RT and other treatment methods has contributed to improved prognosis and pain relief in patients with advanced HCC. The combination of RT and antibodies against programmed cell death protein 1 (anti-PD1) has been effective in patients with advanced HCC, with a median survival of 20.9 months without an increase in treatment side effects (14).

Some studies about patients with HCC who received RT have constructed models to make survival prediction, but studies based on large-scale populations are still needed (15–19). The American National Cancer Institute’s Surveillance, Epidemiology, and End Results (SEER) registry is a publicly available and open database; it records the diagnosis and treatment information of many kinds of cancers. Based on the large-scale populations of the SEER database, we analyzed the long-term survival of patients with HCC with different clinicopathological features. A nomogram model for survival prediction in patients with HCC with RT was created and validated.

## Methods

### Patients and study design

Training and internal validation cohorts: Using SEER*Stat Software (Version 8.4.0), we searched for patients in the SEER 17 registry diagnosed with HCC between 2000 and 2019 to create training and internal validation cohorts. The SEER 17 registry has information, including survival data, for most patients diagnosed with HCC. The information available includes age, sex, T stage, N stage, M stage, histological or clinical grade, tumor number, max tumor size, major vascular invasion (MVI), alpha fetoprotein (AFP), fibrosis, chemotherapy, surgery, vital status, and survival time of the patients.

In the SEER database, the inclusion criteria were patients aged 20 years or older and diagnosed with HCC. The exclusion criteria were patients who did not receive EBRT or without a precise TNM stage. Patients were included in the analysis, although some information other than EBRT conditions or TNM stage were unknown. In our study, the included patients with HCC in the SEER database had complete information about sex, age, TNM stage, chemotherapy, and surgery or not, as well as survival status and survival time. However, other data, including histological or clinical grade, tumor number, max tumor size, MVI, AFP levels, and fibrosis, were unknown in some patients. In **Tables 1** and **2**, a detailed list of unknowns is presented.

**Table 1:** The basic clinicopathological features of patients in the SEER cohorts and external cohorts.

Variable	Overall SEER cohort	SEER training cohort	SEER internal validation cohort	External validation cohort (*n* = 403) (%)
	(*n* = 2,251) (%)	(*n* = 1595) (%)	(*n* = 656) (%)	
Age (years), *n* (%)				
≤50	147 (6.6)	110 (6.9)	37 (5.7)	124 (30.8)
>50, <70	1,455 (64.6)	1,024 (64.2)	431 (65.7)	250 (62.0)
≥70	649 (28.8)	461 (28.9)	188 (28.6)	29 (7.2)
Sex, *n* (%)				
Male	1,845 (82.0)	1,325 (83.1)	520 (79.3)	369 (91.6)
Female	406 (18.0)	270 (16.9)	136 (20.7)	34 (8.4)
T stage, *n* (%)				
T1	912 (40.5)	640 (40.1)	272 (41.5)	85 (21.1)
T2	438 (19.5)	301 (18.9)	137 (20.9)	52 (12.9)
T3	691 (30.7)	507 (31.8)	184 (28.0)	57 (14.1)
T4	210 (9.3)	147 (9.2)	63 (9.6)	209 (51.9)
N stage, *n* (%)				
N0	1,885 (83.7)	1,321 (82.8)	564 (86.0)	321 (79.7)
N1	366 (16.3)	274 (17.2)	92 (14.0)	82 (20.3)
M stage, *n* (%)				
M0	1,136 (50.5)	800 (50.2)	336 (51.2)	348 (86.4)
M1	1,115 (40.5)	795 (49.8)	320 (48.8)	55 (13.6)
Grade, *n* (%)				
Well/moderately	472 (21.0)	350 (21.9)	122 (18.6)	83 (20.6)
Poorly/undifferentiated	193 (8.6)	128 (8.0)	65 (9.9)	93 (23.1)
Unknown	1,586 (70.4)	1,117 (70.1)	469 (71.5)	227 (56.3)
Tumor number, *n* (%)				
Single	628 (27.9)	440 (27.6)	188 (28.7)	174 (43.2)
Multiple	479 (21.2)	340 (21.3)	139 (21.2)	229 (56.8)
Unknown	1,144 (50.9)	815 (51.1)	329 (50.1)	0 (0)
Max tumor size (cm), *n* (%)				
≤2	167 (7.4)	121 (7.7)	46 (7.0)	46 (11.4)
>2, ≤5	793 (35.2)	555 (34.8)	238 (36.3)	115 (28.5)
>5, ≤10	759 (33.7)	537 (33.6)	222 (33.8)	154 (38.2)
>10	310 (13.8)	222 (13.9)	88 (13.4)	88 (21.8)
Unknown	222 (9.9)	160 (10.0)	62 (9.5)	0 (0)
MVI, *n* (%)				
No	1,218 (54.1)	853 (53.5)	365 (55.6)	194 (48.1)
Yes	229 (10.2)	173 (10.8)	56 (8.5)	209 (51.9)
Unknown	804 (35.7)	569 (35.7)	235 (35.9)	0(0)
AFP, *n* (%)				
Negative	345 (15.3)	251 (15.7)	94 (14.3)	162 (40.0)
Positive	1,084 (48.2)	756 (47.4)	328 (50.0)	242 (60.)
Unknown	822 (36.5)	588 (36.9)	234 (35.7)	0 (0)
Fibrosis, *n* (%)				
Yes	389 (17.3)	275 (17.2)	542 (82.8)	227 (56.3)
No/unknown	1,862 (82.7)	1320 (82.8)	114 (17.3)	176 (43.7)
Chemotherapy, *n* (%)				
No	1,182 (52.5)	835 (52.4)	347 (52.9)	127 (31.5)
Yes	1,069 (47.5)	760 (47.6)	309 (47.1)	276 (68.5)
Surgery, *n* (%)				
No	2,039 (90.6)	1,453 (91.1)	586 (89.3)	189 (46.9)
Yes	212 (9.4)	142 (8.9)	70 (10.7)	214 (53.1)

AFP, alpha fetoprotein; MVI, major vascular invasion; SEER, Surveillance, Epidemiology, and End Results; HR, hazard ratio; CI, confidence interval. The data were presented as number of patients (n) and the percentage of the respective group (%).

External validation cohort: We collected patients from Guangxi Medical University Cancer Hospital. The patients who had been diagnosed with HCC between September 2014 and July 2021 were used to create an external validation cohort. The inclusion and exclusion criteria were as follows: patients diagnosed with HCC (based on history and imaging or histopathology), those with liver function of Child–Pugh class A or B, those with an Eastern Cooperative Oncology Group (ECOG) performance score of 0–2, those who received RT and available laboratory tests, and those who were able to complete the treatment plan were included. Patients with combined intrahepatic cholangiocarcinoma and those who lacked laboratory indicators and complete follow-up outcomes were excluded. Ultimately, 403 patients with HCC were included. This study plan was carried out in compliance with the Declaration of Helsinki. The ethics committee gave permission to this retrospective study (ethic no. KYB2024010).

### Statistical analysis

Random number generator by SPSS Statistics software (version 26.0) was adopted to separate the cases from SEER registry into two cohorts. About 70% of the cases in the total sample served as the training set. The remaining 30% of the cases served as the validation set. An additional external validation set was created with the patients from the Guangxi Medical University Cancer Hospital. R and Rstudio software (version 4.3.2) were used to do further survival analysis. Categorical variables were described as frequencies and percentages. The “glmnet” package of R was used to make LASSO regression analysis. The “survival” and “survminer” packages of R were used to do survival analysis. LASSO regression and univariate and multivariate Cox regression analyses were conducted to select the best prognostic factors to be included in, and identify meaningless factors to be excluded for the nomogram. A nomogram model was constructed by the “rms” package of R. The Kaplan–Meier method and log-rank test were used to compare the survival time of patients with different independent factors.

The packages “timeROC” and “foreign” were adopted to plot time-dependent receiver operating characteristic (TROC) curves. Areas under the curve (AUCs) and C-index were counted to evaluate the capability of the nomogram. Decision curve analysis (DCA) by the “ggDCA” package of R and calibration curve by the “tidyr” and “dplyr” packages of R were employed to estimate the clinical effectiveness of the nomogram. All data with *p* < 0.05 were statistically significant.

## Results

### Patients

**Figure 1:**
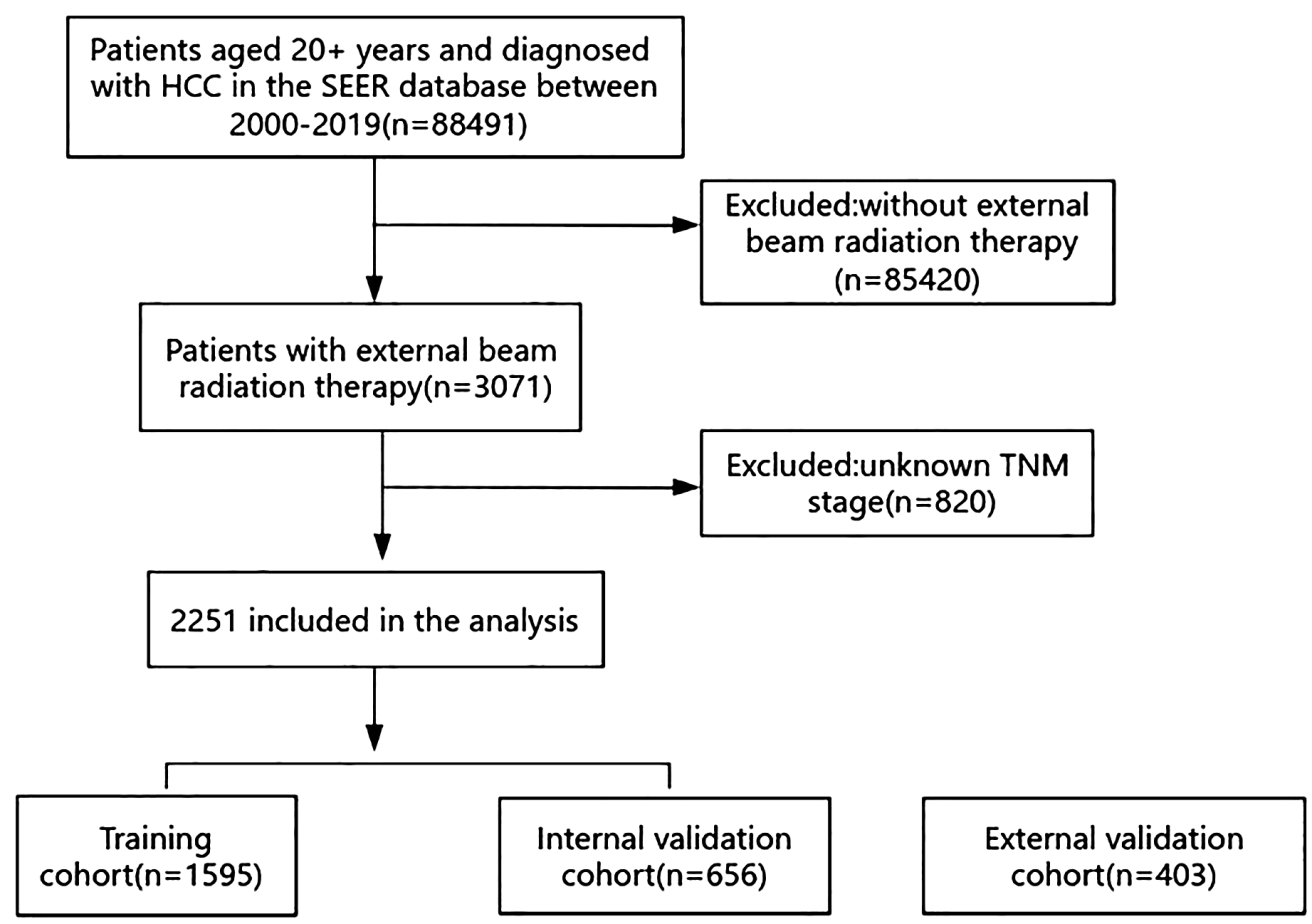
Flowchart for selecting patients from the SEER registry; HCC, hepatocellular carcinoma.

The SEER database included a total of 88,491 patients aged over 20 who had been diagnosed with HCC between 2000 and 2019. After applying the exclusion criteria, 2,251 patients remained. A total of 1,595 patients were randomly assigned to a training cohort and 656 patients were assigned to an internal validation cohort in a 7:3 ratio. The screening process is illustrated in **Figure 1**.

Additionally, an external validation cohort including 403 patients with HCC was from the Guangxi Medical University Cancer Hospital.

The baseline clinical features of all the included patients across the three cohorts are presented in **Table 1**. Most patients (64.6% in the SEER cohort and 62.0% in external validation cohort) were between the ages of 50 and 70; 82.0% of patients in the SEER cohort and 91.6% of patients in the external validation cohort were male. In the SEER cohort, 40.5% of the patients were at the T1 stage, 83.7% were at the N0 stage, and 50.5% were at the M0 stage. In the external validation cohort, 51.9% of the patients were at the T4 stage, 79.7% were at the N0 stage, and 86.4% were at the M0 stage; 47.5% and 68.5% of the patients had undergone chemotherapy in the SEER cohort and external validation cohort, respectively; 9.4% and 53.1% of the patients had undergone surgery in the SEER cohort and external validation cohort, respectively.

### LASSO regression and Cox regression analysis

L1 regularization was incorporated into the LASSO regression to constrain the complexity of the regression model, as it reduces the weight of certain independent variables, potentially reducing them to zero. It is particularly useful for feature selection in cases of variable correlation or collinearity.

**Figure 2:**
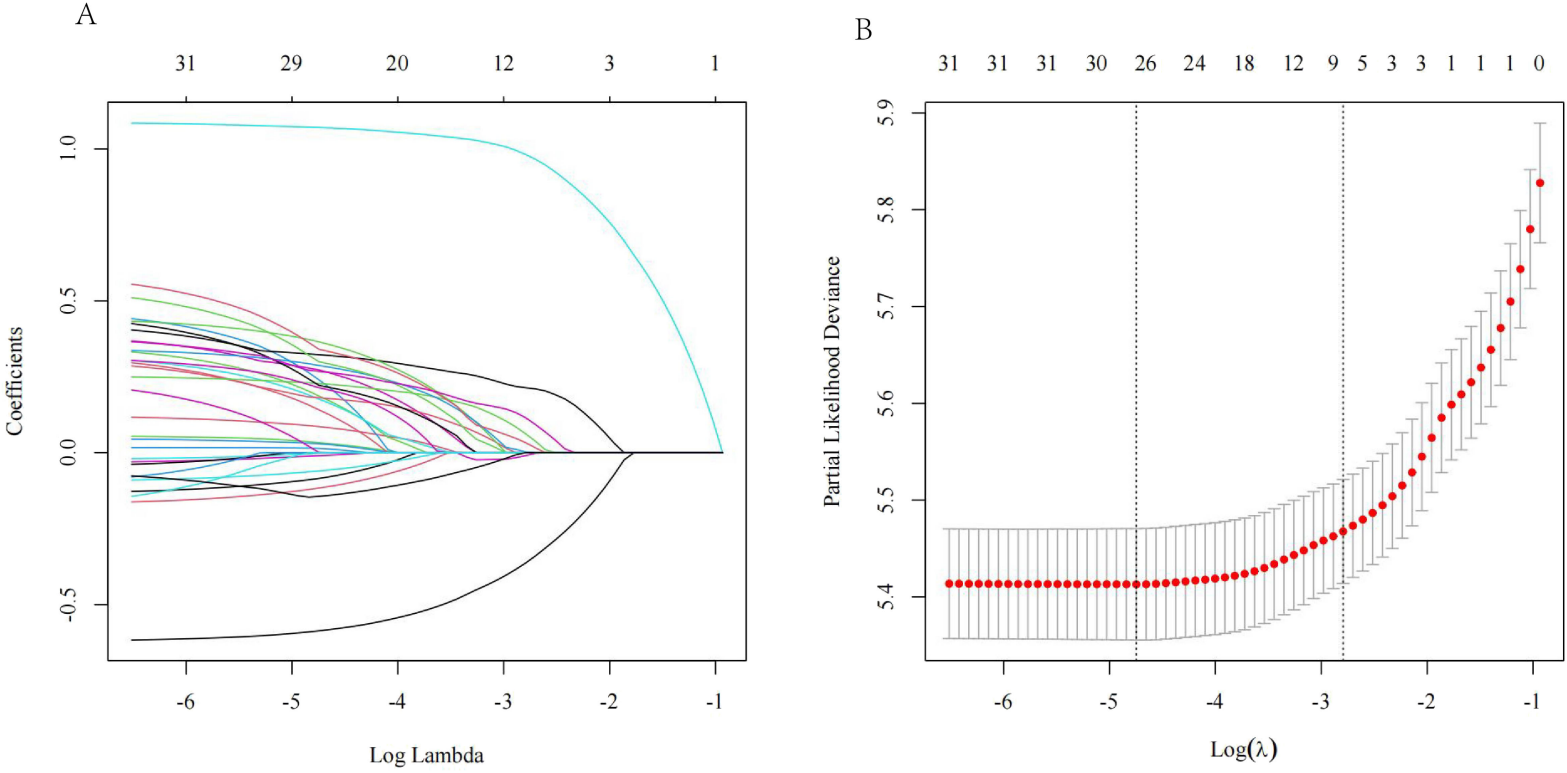
**(A)** Variable coefficient versus l curve of LASSO regression; **(B)** LASSO regression log(l) curve and binomial deviation.

In this study, 13 clinical characteristics were considered for LASSO regression analysis: age, sex, T stage, N stage, M stage, histological or clinical grade, tumor number, tumor size, MVI, AFP, fibrosis, chemotherapy, and surgery. The analysis revealed that the non-zero coefficients corresponded to T stage, N stage, M stage, tumor size, MVI, AFP, and chemotherapy in the training group (**Figure 2**).

### Univariate and multivariate analyses

In the SEER training cohort, 13 prognostic factors—age, sex, T stage, N stage, M stage, histological or clinical grade, tumor number, max tumor size, MVI, AFP, fibrosis, chemotherapy, and surgery—were evaluated through univariate Cox regression analyses. The significant variables were T stage, N stage, M stage, tumor number, max tumor size, grade, MVI, AFP, fibrosis, and chemotherapy. The multivariate analysis was further analyzed, and the results showed that the significant variables were T stage {T2 vs. T1, hazard ratio (HR) = 1.452 [95% CI, 1.195–1.765], *p* < 0.001; T3 vs. T1, HR = 1.469 [95% CI, 1.168–1.846], *p* < 0.001; T4 vs. T1, HR = 1.291 [95% CI, 0.951–1.754], *p* = 0.101}, N stage (HR = 1.555 [95% CI, 1.338–1.805], *p* < 0.001), M stage (HR = 3.007 [95% CI, 2.645–3.418], *p* < 0.001), max tumor size (>2 and ≤5 vs. ≤2 cm, HR = 1.273 [95% CI, 0.992–1.633], *p* = 0.057; >5 and ≤10 vs. ≤2 cm, HR = 1.625 [95% CI, 1.246–2.118], *p* < 0.001; >10 vs. ≤2 cm, HR = 1.784 [95% CI, 1.335–2.385], *p* < 0.001), MVI (HR = 1.454 [95% CI, 1.028–2.057], *p* = 0.034), AFP (HR = 1.573 [95% CI, 1.315–1.882], *p* < 0.001), and chemotherapy (HR = 0.511 [95% CI, 0.454–0.576], *p* < 0.001), which agreed with the results of the LASSO regression analysis (**Table 2**).

**Table 2:** Cox analyses of the clinicopathological variables.

Variable	Univariable analysis	*p*-value	Multivariable analysis	*p*-value
	HR (95% CI)		HR (95% CI)	
Age (years)				
(>50, <70 vs. ≤50)	0.989 (0.794–1.232)	0.925		
(≥70 vs. ≤50)	1.047 (0.830–1.321)	0.697		
Sex (female vs. male)	0.911 (0.782–1.061)	0.231		
T stage (T2 vs.T1)	1.181 (1.009–1.383)	0.037	1.452 (1.195–1.765)	<0.001
(T3 vs.T1)	2.024 (1.775– 2.307)	<0.001	1.469 (1.168–1.846)	<0.001
(T4 vs.T1)	1.984 (1.613–2.441)	<0.001	1.291 (0.951–1.754)	0.101
N stage (N1 vs.N0)	1.741 (1.477–2.053)	<0.001	1.555 (1.338–1.805)	<0.001
M stage (M1 vs.M0)	3.225 (2.868–3.625)	<0.001	3.007 (2.645–3.418)	<0.001
Grade(poorly/undifferentiated vs. well/moderately)	1.374 (1.099–1.719)	0.005	1.227 (0.976–1.541)	0.141
Tumor number (multiple vs. single)	1.496 (1.289–1.736)	<0.001	0.895 (0.714–1.122)	0.337
Max tumor size (cm)				
(>2, ≤5 vs. ≤2)	1.428 (1.119–1.823)	0.004	1.273 (0.992–1.633)	0.057
(>5, ≤10 vs. ≤2)	2.362 (1.851–3.013)	<0.001	1.625 (1.246–2.118)	<0.001
(>10 vs. ≤2)	2.916 (2.232–3.809)	<0.001	1.784 (1.335–2.385)	<0.001
MVI (yes vs. no)	1.570 (1.320–1.867)	<0.001	1.454 (1.028–2.057)	0.034
AFP (positive vs. negative)	1.823 (1.528–2.175)	<0.001	1.573 (1.315–1.882)	<0.001
Fibrosis (yes vs. no/unknown)	0.797 (0.684–0.929)	0.003	0.918 (0.782–1.077)	0.295
Chemotherapy (yes vs. no)	0.737 (0.660–0.823)	<0.001	0.511 (0.454–0.576)	<0.001
Surgery (yes vs. no)	0.973 (0.801–1.183)	0.787		

AFP, alpha fetoprotein; MVI, major vascular invasion; HR, hazard ratio, CI, confidence interval.

**Figure 3:**
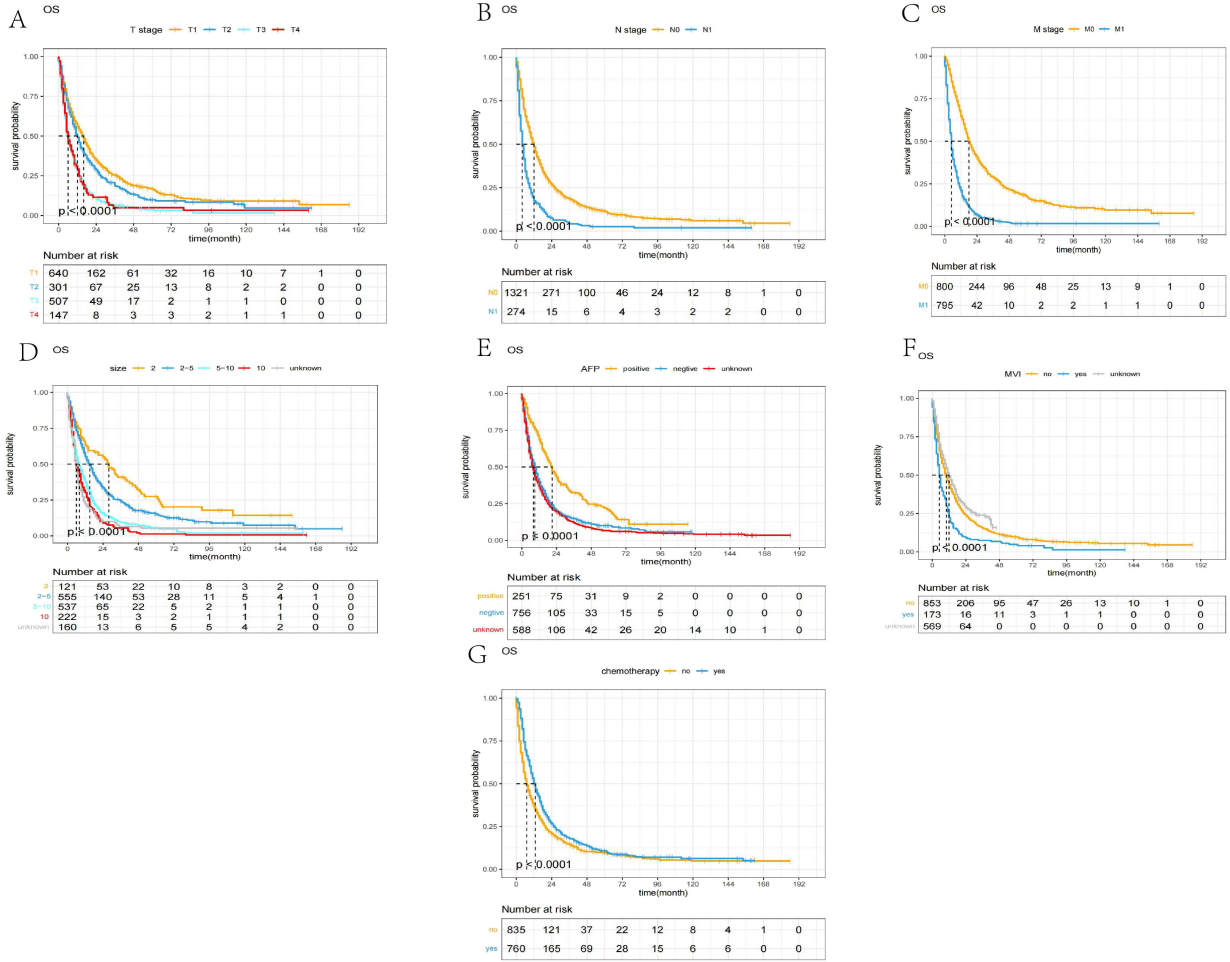
The Kaplan–Meier curves of OS between different groups. **(A)** T stage; **(B)** N stage; **(C)** M stage; **(D)** max tumor size; **(E)** AFP, alpha fetoprotein; **(F)** MVI, major vascular invasion; **(G)** chemotherapy.

The Kaplan–Meier curves based on T stage, N stage, M stage, max tumor size, MVI, AFP, and chemotherapy are shown in **Figure 3**.

### The construction and interpretation of the nomogram

**Figure 4:**
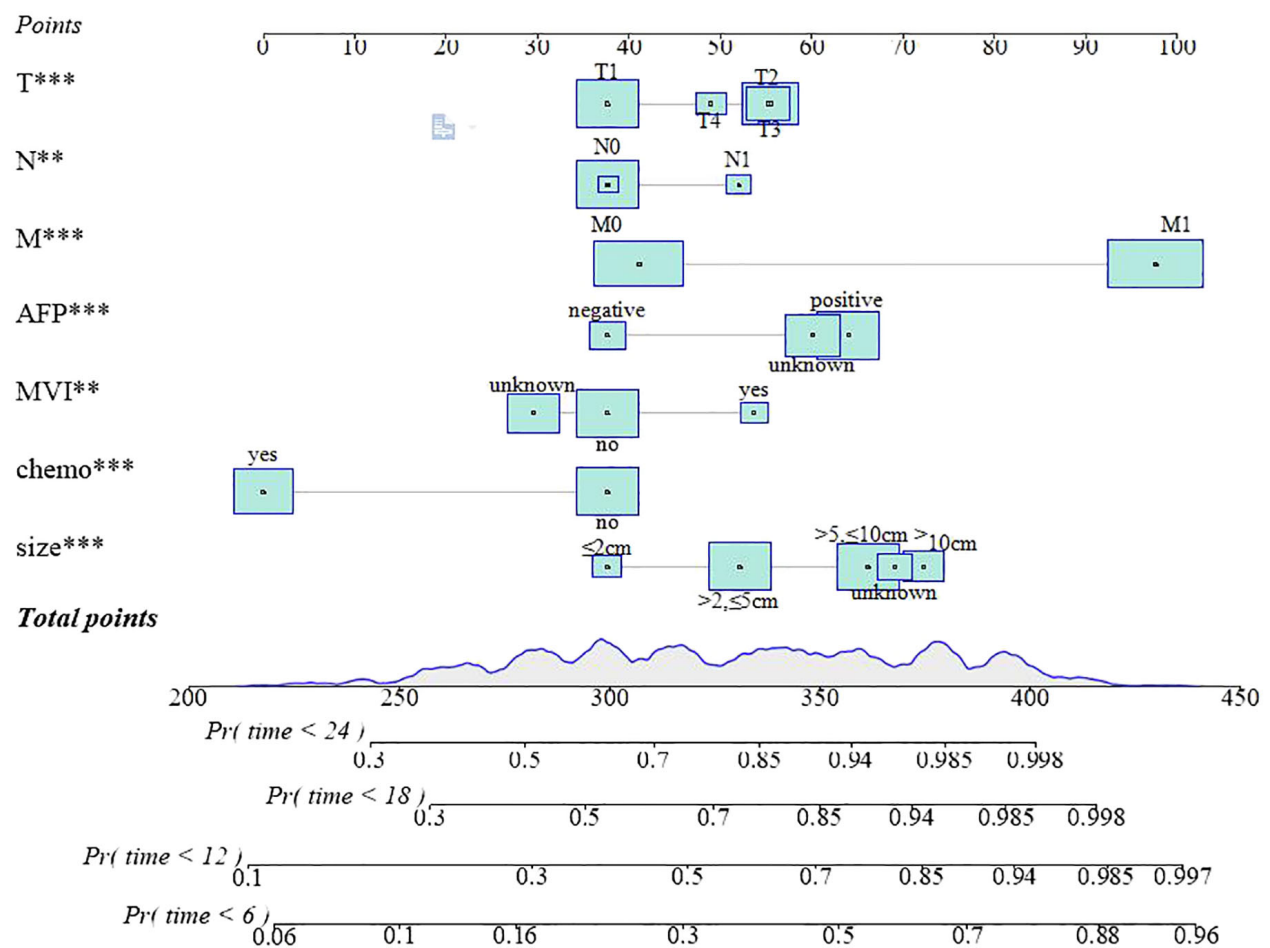
The nomogram predicted the OS rates of 6, 12, 18, and 24 months. T***, T stage; N**, N stage; M***, M stage; AFP***, alpha fetoprotein;MVI**, major vascular invasion; chemo***, chemotherapy; size***, max tumor size.

A nomogram prediction model was developed, according to the significance level of *p* < 0.05 in the multivariate Cox analysis aligned with LASSO regression. By counting the total scores based on the bottom probability column, the OS rates of different time points of patients with HCC undergoing RT were derived (**Figure 4**).

### The clinical utility and accuracy of the nomogram

**Figure 5:**
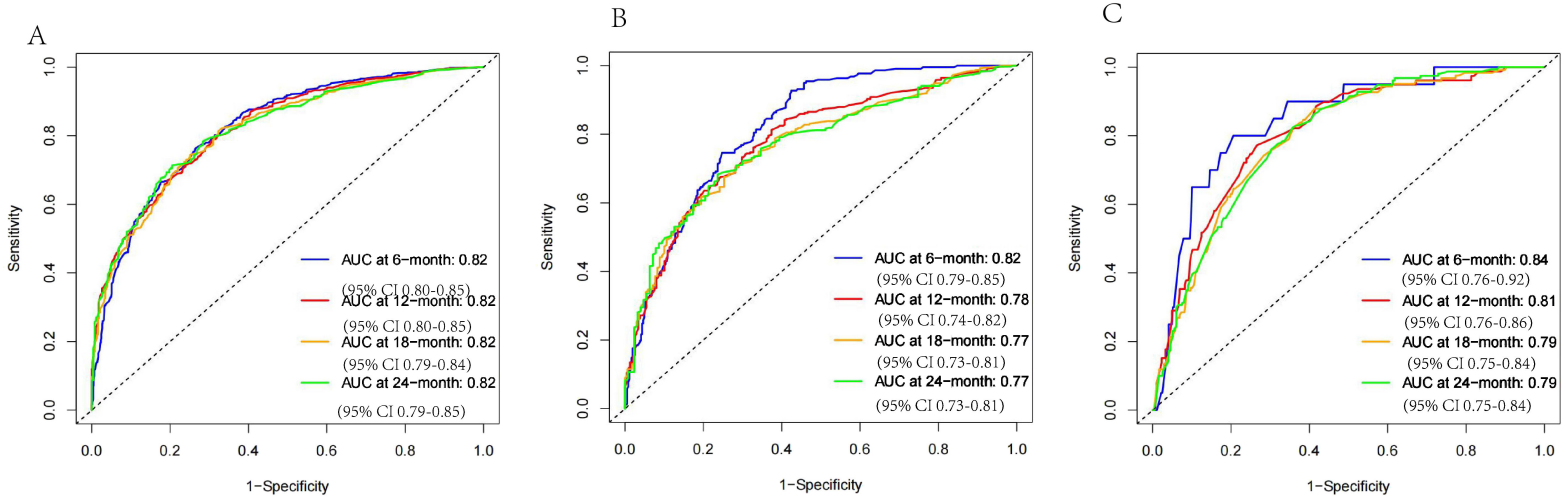
The ROC curves of **(A)** the SEER training cohort; **(B)** the SEER internal validation cohort; and **(C)** the external validation cohort.

For the patients with HCC treated with RT, a nomogram to make survival prediction at different time points was constructed. Two validation cohorts were adopted to value the model, an internal validation cohort (*n* = 656) from the SEER registrar and a cohort (*n* = 403) of Chinese patients. The TROC curves showed the accuracy of the model (**Figure 5**). The AUCs for predicting different time points of 6-, 12-, 18-, and 24-month OS rates were calculated. The AUCs were 0.824 (95% CI, 0.803–0.846), 0.824 (95% CI, 0.802–0.845), 0.816 (95% CI, 0.792–0.840), and 0.820 (95% CI, 0.794–0.846) in the training cohort, respectively. In the internal validation cohort, the AUCs were 0.821 (95% CI, 0.788–0.854), 0.780 (95% CI, 0.743–0.818), 0.769 (95% CI, 0.729–0.809), and 0.769 (95% CI, 0.726–0.813), respectively. In the external validation cohort, the AUCs were 0.844 (95% CI, 0.764–0.924), 0.807 (95% CI, 0.756–0.859), 0.792 (95% CI, 0.746–0.839), and 0.793 (95% CI, 0.747–0.839), respectively. The C-index are 0.701, 0.697, and 0.694 for the training cohort, internal validation cohort, and external validation cohort, respectively.

**Figure 6:**
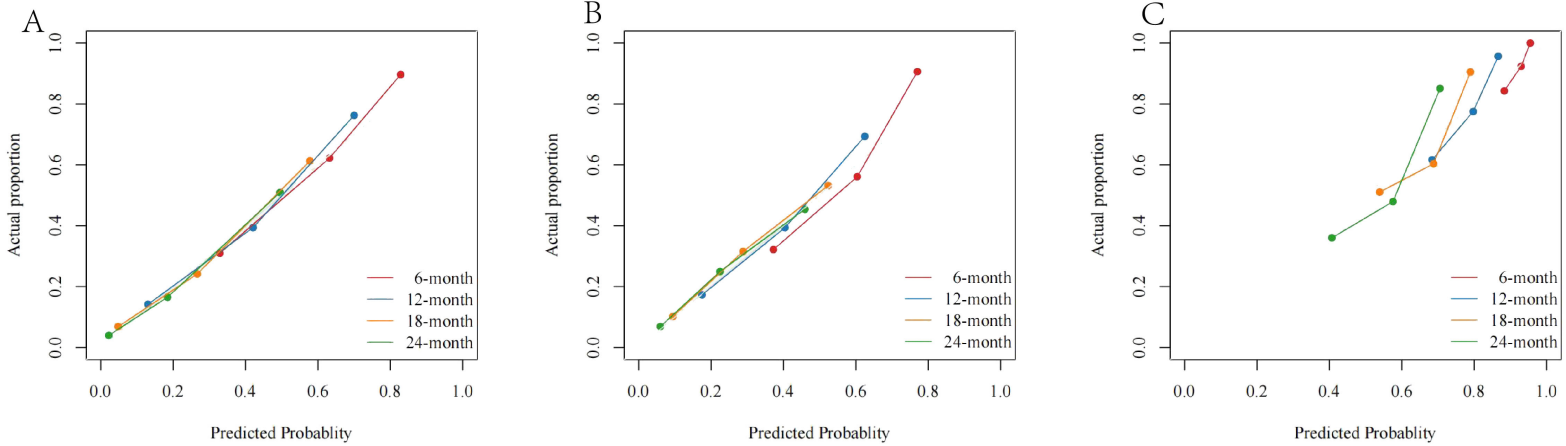
The calibration curves of **(A)** the SEER training cohort; **(B)** the SEER internal validation cohort; and **(C)** the external cohort.

The calibration curves showed that the difference between the actual value and the predicted value was relatively small (**Figure 6**). This result further confirms the applicability of the model in accurately predicting the OS rates.

**Figure 7:**
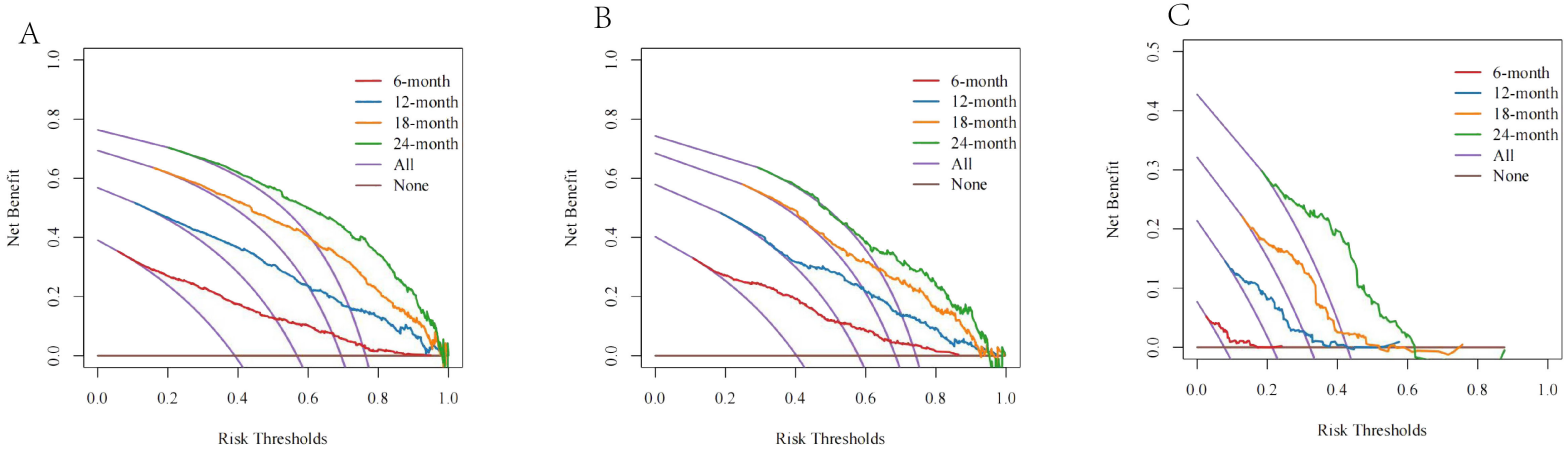
The DCA curves of **(A)** the SEER training cohort; **(B)** the SEER internal validation cohort; and **(C)** the external cohort.

The DCA curve showed that the net benefit rate was higher than that of the extreme curve in a large range of horizontal coordinates, indicating that the DCA curve has certain application value (**Figure 7**). The DCA curve suggested good clinical prediction; at the same time, the DCA curve had a high benefit.

## Discussion

The new diagnosis of HCC has increased approximately 4%–6% every year over the last decade, emphasizing the significant influence of HCC on public health, from records of the National Cancer Institute (20). The prognosis of HCC is poor, because of the late detection and the malignancy of the tumor. The treatment of advanced HCC can be tough. With improving RT techniques, RT has gradually become an important part of HCC treatment, whether it is radical treatment or palliative treatment (21). Therefore, the ability to stratify patients based on their potential for benefit from RT is important. Although there have been some studies that have established nomogram models to predict the prognosis, a large-scale population study of survival prediction is still needed for patients with HCC who received RT (15–19).

We chose 13 variables for analysis, namely, age, sex, T stage, N stage, M stage, histological or clinical grade, tumor number, max tumor size, MVI, AFP, fibrosis, chemotherapy, and surgery. By using the LASSO regression, independent variables were better analyzed, and the most influential variables were chosen.

The Cox regression analyses identified seven independent risk factors. A nomogram constructed with T stage, N stage, M stage, max tumor size, MVI, AFP, and chemotherapy has good survival prediction ability, for patients with HCC who had received RT. The nomogram was validated through two validation cohorts. Based on the results of the AUC and C-index, the model performed well in predicting OS. The DCA and calibration curve also showed the accuracy and reliability for survival prediction of the model. Using the nomogram, the individualized OS can be estimated easily.

The American Joint Committee on Cancer (AJCC) TNM staging (eighth edition, 2017) is currently the most widely utilized clinical reference for the treatment decision of all kinds of cancers (22). The Barcelona Clinic Liver Cancer system and AJCC TNM stages are commonly employed clinical staging methods for HCC. It is generally acknowledged that the advanced TNM stage means a worse prognosis (23).

Furthermore, the AFP levels in peripheral venous blood have been extensively used for the diagnosis and monitoring of HCC. Clinical observations have indicated that AFP-positive patients with HCC exhibit high malignancy, rapid progression, and poor prognoses compared to those with AFP-negative HCC. In the context of liver transplantation, AFP is also recognized as a marker of HCC biology, and clinical trials have been conducted on a cancer vaccine utilizing an AFP-derived class I-restricted epitope as the antigen (24). Similarly, our study demonstrated that AFP-negative patients with HCC had a more favorable prognosis than AFP-positive individuals.

In the treatment and management of advanced HCC, the therapy of palliative chemotherapy, particularly oxaliplatin-based systemic chemotherapy, is considered crucial (25). The combination of bevacizumab with gemcitabine and oxaliplatin has demonstrated enhanced efficacy, with a progression-free survival (PFS) of 70% (95% CI, 54%–85%) at 3 months and 48% (95% CI, 31%–65%) at 6 months, and a median OS of 9.6 months for patients with advanced HCC (26).

Similarly, hepatic arterial infusion of fluorouracil and oxaliplatin can greatly prolong OS for patients with HCC with unresectable large tumor, according to the report of a recent randomized phase III trial (27). Our research also suggested that the combination therapy of chemotherapy with RT led to a favorable prognosis for patients with HCC. The analysis revealed that patients who received RT and chemotherapy exhibited a significantly longer survival time.

The portal hypertension and MVI in HCC are strongly correlated with a bad survival prognosis. Patients with HCC with MVI typically exhibit a shorter median survival time of approximately 2.7–4.0 months and have limited treatment options (28). RT has been identified as an effective locoregional treatment for patients with HCC with MVI (13). The combination of transcatheter arterial chemoembolization(TACE) and RT can improve the prognosis of patients with HCC with MVI (29).

Tumor size in HCC is a significant predictor of survival, with larger tumors being associated with higher rates of recurrence, poor cancer-specific survival, and increased regional and distant metastases. Tumor size is considered one of the most reliable markers of aggressiveness in HCC (30). In our study, it was observed that patients with MVI or larger tumor size experienced a poor prognosis.

Compared to other studies, our research relies on the American public database, selected a large sample population to build a model, and verifies the model in the Chinese population. The selected factors are comprehensive and easy to obtain clinically. We hope to make aware of the important role of RT and provide some references for the physician in making decision of HCC treatment. The combination of RT and anti-PD1 has been effective in patients with advanced HCC, with a median survival of 20.9 months without an increase in treatment side effects (31–33). RT has the ability to transform the tumor microenvironment, characterized by low immunogenicity and inadequate immune cell infiltration, into one with a robust immunological response (34, 35). The immune system activated by RT can also be used in conjunction with immune checkpoint inhibitors (ICIs) to disrupt immune escape and produce a more potent antitumor effect. Sequential RT after progression during anti-PD1 therapy in advanced HCC resulted in a median OS of 18.8 months and a median PFS of 7.4 months (36). These findings confirm the significant role of RT and combination therapy in the treatment of HCC.

One controversial result in our research is that surgery was not statistically significant on the survival prediction of patients with HCC with RT in the SEER database. Both the LASSO regression and multivariate analysis confirmed this point. In the SEER database, the proportion of patients who received both EBRT and surgery was relatively small, which may affect the analysis results. Furthermore, the patients who received both surgery and RT were mostly in the advanced stage; thus, they may have experienced significant side effects and potentially died from treatment-related adverse reactions. A study conducted by Wen-Yen Huang et al. in 2020 also found that whether patients received surgery or not before radiotherapy had no difference in their prognosis (19). Therefore, we need to comprehensively assess the patient’s condition, including staging, general health, liver function, underlying diseases, and economic status to develop the most favorable treatment plan.

Although our model had good clinical predictive power, it was limited by some shortcomings. As a retrospective clinical study, it had selection and information biases. We lacked clinical data, such as liver function tests and coagulation function, which are very important in evaluating treatment options and efficacy. We also lacked specific therapy information, such as the RT dose, drug regimen, and the provision of radiofrequency ablation or TACE. Furthermore, there were no records to determine if patients had comorbidities, such as hepatitis, fatty liver disease, diabetes, or other pathologies that may have affected their prognosis.

## Conclusion

In conclusion, for patients with HCC treated with RT, a nomogram constructed with T stage, N stage, M stage, max tumor size, MVI, AFP, and chemotherapy has good survival prediction ability. Our model provides a reference for clinicians to make decisions on HCC treatment. RT plays an important role in the comprehensive treatment of patients with HCC, and more clinical and molecular mechanism studies are needed in the future to focus on improving the efficacy of RT for HCC.

## Data Availability

The raw data supporting the conclusions of this article will be made available by the authors, without undue reservation.

## References

[1] Sung H Ferlay J Siegel RL Laversanne M Soerjomataram I Jemal A. Global cancer statistics 2020: GLOBOCAN estimates of incidence and mortality worldwide for 36 cancers in 185 countries. CA Cancer J Clin. (2021) 71:209–49. doi: 10.3322/caac.21660 33538338

[2] Anwanwan D Singh SK Singh S Saikam V Singh R. Challenges in liver cancer and possible treatment approaches. Biochim Biophys Acta Rev Cancer. (2020) 1873:188314. doi: 10.1016/j.bbcan.2019.188314 31682895 PMC6981221

[3] Horvat N de Oliveira AI Clemente de Oliveira B Araujo-Filho JAB El Homsi M Elsakka A. Local-regional treatment of hepatocellular carcinoma: A primer for radiologists. Radiographics. (2022) 42:1670–89. doi: 10.1148/rg.220022 PMC9539394 36190854

[4] Vibert E Schwartz M Olthoff KM. Advances in resection and transplantation for hepatocellular carcinoma. J Hepatol. (2020) 72:262–76. doi: 10.1016/j.jhep.2019.11.017 31954491

[5] Park KW Park JW Choi JI Kim TH Kim SH Park HS. Survival analysis of 904 patients with hepatocellular carcinoma in a hepatitis B virus-endemic area. J Gastroenterol Hepatol. (2008) 23:467–73. doi: 10.1111/j.1440-1746.2007.05112.x 17764529

[6] Bae SH Chun SJ Chung JH Kim E Kang JK Jang WI. Stereotactic body radiation therapy for hepatocellular carcinoma: meta-analysis and international stereotactic radiosurgery society practice guidelines. Int J Radiat Oncol Biol Phys. (2023). doi: 10.1016/j.ijrobp.2023.08.015 37597757

[7] Marrero JA Kulik LM Sirlin CB Zhu AX Finn RS Abecassis MM. Diagnosis, staging, and management of hepatocellular carcinoma: 2018 practice guidance by the american association for the study of liver diseases. Hepatology. (2018) 68:723–50. doi: 10.1002/hep.29913 29624699

[8] Bujold A Massey CA Kim JJ Brierley J Cho C Wong RK. Sequential phase I and II trials of stereotactic body radiotherapy for locally advanced hepatocellular carcinoma. J Clin Oncol. (2013) 31:1631–9. doi: 10.1200/JCO.2012.44.1659 23547075

[9] Liang SX Zhu XD Lu HJ Pan CY Li FX Huang QF. Hypofractionated three-dimensional conformal radiation therapy for primary liver carcinoma. Cancer. (2005) 103:2181–8. doi: 10.1002/cncr.21012 15812834

[10] Benson AB D'Angelica MI Abbott DE Anaya DA Anders R Are C. Hepatobiliary cancers, version 2.2021, NCCN clinical practice guidelines in oncology. J Natl Compr Canc Netw. (2021) 19:541–65. doi: 10.6004/jnccn.2021.0022 34030131

[11] Son SH Choi BO Ryu MR Kang YN Jang JS Bae SH. Stereotactic body radiotherapy for patients with unresectable primary hepatocellular carcinoma: dose-volumetric parameters predicting the hepatic complication. Int J Radiat Oncol Biol Phys. (2010) 78:1073–80. doi: 10.1016/j.ijrobp.2009.09.009 20207492

[12] Li JX Zhang RJ Qiu MQ Yan LY He ML Long MY. Non-classic radiation-induced liver disease after intensity-modulated radiotherapy for Child-Pugh grade B patients with locally advanced hepatocellular carcinoma. Radiat Oncol. (2023) 18:48. doi: 10.1186/s13014-023-02232-5 36890592 PMC9993633

[13] Li LQ Zhou Y Huang Y Liang P Liang SX Su TS. Stereotactic body radiotherapy versus intensity-modulated radiotherapy for hepatocellular carcinoma with portal vein tumor thrombosis. Hepatol Int. (2021) 15:630–41. doi: 10.1007/s12072-021-10173-y 33818714

[14] Li JX Su TS Gong WF Zhong JH Yan LY Zhang J. Combining stereotactic body radiotherapy with camrelizumab for unresectable hepatocellular carcinoma: a single-arm trial. Hepatol Int. (2022) 16:1179–87. doi: 10.1007/s12072-022-10396-7 PMC9525355 36001228

[15] Zhan G Peng H Zhou L Jin L Xie X He Y. A web-based nomogram model for predicting the overall survival of hepatocellular carcinoma patients with external beam radiation therapy: A population study based on SEER database and a Chinese cohort. Front Endocrinol (Lausanne). (2023) 14:1070396. doi: 10.3389/fendo.2023.1070396 36798659 PMC9927006

[16] Hua Q Zhang D Li Y Hu Y Liu P Xiao G. Prognostic factors of survival of advanced liver cancer patients treated with palliative radiotherapy: A retrospective study. Front Oncol. (2021) 11:658152. doi: 10.3389/fonc.2021.658152 34395242 PMC8355619

[17] Li X Ye Z Lin S Pang H. Predictive factors for survival following stereotactic body radiotherapy for hepatocellular carcinoma with portal vein tumour thrombosis and construction of a nomogram. BMC Cancer. (2021) 21:701. doi: 10.1186/s12885-021-08469-1 34126955 PMC8204556

[18] Long M Li J He M Qiu J Zhang R Liu Y. Establishment and validation of a prognostic pomogram in unresectable hepatocellular carcinoma treated with intensity modulated radiotherapy: a real world study. Radiat Oncol. (2023) 18:96. doi: 10.1186/s13014-023-02292-7 37287040 PMC10245442

[19] Huang WY Tsai CL Que JY Lo CH Lin YJ Dai YH. Development and validation of a nomogram for patients with nonmetastatic BCLC stage C hepatocellular carcinoma after stereotactic body radiotherapy. Liver Cancer. (2020) 9:326–37. doi: 10.1159/000505693 PMC7325119 32647634

[20] Rawla P Sunkara T Muralidharan P Raj JP. Update in global trends and aetiology of hepatocellular carcinoma. Contemp Oncol (Pozn). (2018) 22:141–50. doi: 10.5114/wo.2018.78941 PMC6238087 30455585

[21] Yan K. Recent developments in radiotherapy. N Engl J Med. (2017) 377:2200. doi: 10.1056/NEJMc1713349 29188983

[22] Subramaniam S Kelley RK Venook AP. A review of hepatocellular carcinoma (HCC) staging systems. Chin Clin Oncol. (2013) 2:33. doi: 10.3978/j.issn.2304-3865.2013.07.05 25841912

[23] Bruix J Reig M Sherman M. Evidence-based diagnosis, staging, and treatment of patients with hepatocellular carcinoma. Gastroenterology. (2016) 150:835–53. doi: 10.1053/j.gastro.2015.12.041 26795574

[24] Hu X Chen R Wei Q Xu X.. The landscape of alpha fetoprotein in hepatocellular carcinoma: where are we? Int J Biol Sci. (2022) 18:536–51. doi: 10.7150/ijbs.64537 PMC8741863 35002508

[25] Petrelli F Coinu A Borgonovo K Cabiddu M Ghilardi M Lonati V. Oxaliplatin-based chemotherapy: a new option in advanced hepatocellular carcinoma. a systematic review and pooled analysis. Clin Oncol (R Coll Radiol). (2014) 26:488–96. doi: 10.1016/j.clon.2014.04.031 24856442

[26] Zhu AX Blaszkowsky LS Ryan DP Clark JW Muzikansky A Horgan K. Phase II study of gemcitabine and oxaliplatin in combination with bevacizumab in patients with advanced hepatocellular carcinoma. J Clin Oncol. (2006) 24:1898–903. doi: 10.1200/JCO.2005.04.9130 16622265

[27] Li QJ He MK Chen HW Fang WQ Zhou YM Xu L. Hepatic arterial infusion of oxaliplatin, fluorouracil, and leucovorin versus transarterial chemoembolization for large hepatocellular carcinoma: A randomized phase III trial. J Clin Oncol. (2022) 40:150–60. doi: 10.1200/JCO.21.00608 34648352

[28] Minagawa M Makuuchi M. Treatment of hepatocellular carcinoma accompanied by portal vein tumor thrombus. World J Gastroenterol. (2006) 12:7561–7. doi: 10.3748/wjg.v12.i47.7561 PMC4088035 17171782

[29] Duan F Yu W Wang Y Liu FY Song P Wang ZJ. Trans-arterial chemoembolization and external beam radiation therapy for treatment of hepatocellular carcinoma with a tumor thrombus in the inferior vena cava and right atrium. Cancer Imaging. (2015) 15:7. doi: 10.1186/s40644-015-0043-3 26007646 PMC4488985

[30] Usta S Kayaalp C. Tumor diameter for hepatocellular carcinoma: why should size matter? J Gastrointest Cancer. (2020) 51:1114–7. doi: 10.1007/s12029-020-00483-z 32851543

[31] Li JX Deng WX Huang ST Lin XF Long MY Zhang J. Efficacy and safety of radiotherapy plus anti-PD1 versus transcatheter arterial chemoembolization plus sorafenib for advanced hepatocellular carcinoma: a real-world study. Radiat Oncol. (2022) 17:106. doi: 10.1186/s13014-022-02075-6 35690773 PMC9188229

[32] Zhang RJ Zhou HM Lu HY Yu HP Tang WZ Qiu MQ. Radiotherapy plus anti-PD1 versus radiotherapy for hepatic toxicity in patients with hepatocellular carcinoma. Radiat Oncol. (2023) 18:129. doi: 10.1186/s13014-023-02309-1 37542246 PMC10403970

[33] Hsu S Chao Y Hu Y Zhang Y Hong W Chen Y. Radiotherapy enhances efficacy of PD-1 inhibitors in advanced hepatocellular carcinoma: A propensity-matched real-world study. Chin Med J (Engl). (2024). doi: 10.1097/CM9.0000000000003124 PMC11191029 38725345

[34] Bernstein MB Krishnan S Hodge JW Chang JY. Immunotherapy and stereotactic ablative radiotherapy (ISABR): a curative approach? Nat Rev Clin Oncol. (2016) 13:516–24. doi: 10.1038/nrclinonc.2016.30 PMC6053911 26951040

[35] Chami P Diab Y Khalil DN Azhari H Jarnagin WR Abou-Alfa GK. Radiation and immune checkpoint inhibitors: combination therapy for treatment of hepatocellular carcinoma. Int J Mol Sci. (2023) 24:23. doi: 10.3390/ijms242316773 PMC10706661 38069095

[36] Ning C Jia J Zhang X Sun J Wang Y Xue J. Efficacy and safety of subsequent radiotherapy in patients with advanced-stage hepatocellular carcinoma treated with immune checkpoint inhibitors. Hepatobiliary Surg Nutr. (2023) 12:882–97. doi: 10.21037/hbsn PMC10727817 38115944

